# A Summary Report of FSCJ Workshop “Future Challenges and Opportunities in Developing Methodologies for Improved Human Risk Assessments”

**DOI:** 10.14252/foodsafetyfscj.2018017

**Published:** 2019-11-22

**Authors:** Kaoruko Tachibana, George E.N. Kass, Atsushi Ono, Takashi Yamada, Weida Tong, Daniel R. Doerge, Yasushi Yamazoe

**Affiliations:** 1 Department of Environmental Medicine, Kochi Medical School, Kohasu, Oko-cho, Nankoku-shi, Kochi 783-8505, Japan; 2 Food Safety Commission of Japan, Cabinet Office, Government of Japan, Akasaka Park Bldg, 22F, 5-2-20 Akasaka, Minatoku, Tokyo 107-6122, Japan; 3 European Food Safety Authority, Via Carlo Magno 1A, 43126 Parma, Italy; 4 Okayama University Graduate School of Medicine, Dentistry and Pharmaceutical Sciences, 1-1-1 Tsushimanaka Kita-ku, Okayama-shi, Okayama 700-8530, Japan; 5 National Institute of Health Sciences, 3-25-26 Tonomachi, Kawasaki-shi, Kanagawa 210-0821, Japan; 6 National Center for Toxicological Research, Food and Drug Administration, 3900 NCTR Road, Jefferson, AR 72079, United States of America

**Keywords:** risk assessment methodologies, food safety risk assessments, TTC, read-across, toxicological databases, PBPK modeling

## Abstract

This is a summary report of FSCJ (Food Safety Commission of Japan) workshop entitled “Future Challenges and Opportunities in Developing Methodologies for Improved Human Risk Assessments, which held in November 2018. Scientific advancements have facilitated the development of new methods for chemical risk assessments with the expansion of toxicological databases. They are promising tools to overcome challenges, such as situations of data insufficiency, estimation of internal exposure and prediction of hazard, and enable us to improve our human health risk assessment in food safety. In this review, current understandings on developments in chemical risk assessments, especially focusing on Threshold of Toxicological Concern (TTC) approach, non-testing and *in-silico* approaches (e.g. read-across), and physiologically based pharmacokinetics (PBPK) modeling are discussed as possible promising tools. It also discusses future challenges and opportunities regarding social environment buildings in which all stakeholders including scientific experts, risk managers and consumers are able to accept these new risk assessment technologies. International collaboration would increase and enhance the efficiency in forming innovative ideas and in translating them into regulatory practices. It would strengthen technical capacity of experts who contribute to regulatory decisions and also promote acceptance of new methodologies among stakeholders. Cross-sectional collaboration such as making good use of human data of pharmaceutical drugs will facilitate a development of fresh tools for food safety domains. Once a new methodology is recognized in risk assessment agencies as implementable, it needs to be acknowledged and accepted by wider range of different stakeholders. Such stakeholders include scientific experts who conduct risk assessment for the risk assessment agencies, food industries and consumers. Transparency in the risk assessment work performed by regulatory agencies should strengthen their credibility and promote the acceptance of risk assessment including the new methodologies used in it. At the same time, efforts should be continued by regulatory agencies to further communicate with consumers about the concept of risk-based assessment as well as the concept of uncertainty.

## 1. Background

Since its establishment in 2003, Food Safety Commission of Japan (FSCJ) has conducted science-based risk assessments of chemical and microbiological hazards in foods, using the most appropriate and reliable devices available for risk assessments. Scientific advancements and the expansion of toxicological databases have facilitated the progress in the development of new methods and tools to overcome hurdles in chemical risk assessments, such as scarce data and uncertainties caused by inter-species differences. Given this background, FSCJ held a technical workshop in November 2018 in Tokyo, with experts from Japan, EU and USA to present current status of development and discuss future challenges toward further development and introduction of their new risk assessment methodologies into practice. The workshop has provided a good opportunity for participants to consider how best we can make advantage of the asset of accumulated knowledge and experiences in Japan and globally, for a better human health risk assessment in food safety.

## 2. Objectives

Based on the presentations and discussions at the workshop, this paper aims to provide current understanding of developments in chemical risk assessment, especially focusing on Threshold of Toxicological Concern (TTC) approach, non-testing and *in-silico* approaches (e.g. read-across) and physiologically based pharmacokinetics (PBPK) modeling. This paper also discusses future challenges regarding a building of social environments in which all stakeholders including scientific experts, risk managers and consumers are able to accept these new risk assessment methods.

## 3. Overcoming Data Gaps by Making Use of Existing Data

### 3-1 Use of TTC and Human Relevance

TTC approach is a screening and prioritization tool for the safety assessment of substances, which chemical-structures are known but the hazard data are incomplete and the human exposure can be estimated. The TTC approach uses threshold values as representing life-long human exposure thresholds. The basis to the TTC approach is that exposure below the corresponding threshold values is considered of negligible probability of adverse health effects.

The application of the TTC concept utilizes the classification scheme for chemicals which was originally proposed in late 70’s by Cramer^1^^)^ as a priority setting tool and as a means to make expert judgments in food chemical safety assessment more transparent and reproducible. In brief, the criteria they proposed for the three structural classes are as follows and are based on a set of 33 questions used for classification: Class I stands for substances with simple chemical structures and for which efficient modes of metabolism exist, suggesting a low order of oral toxicity; Class II is for substances that possess structures that are less innocuous than class I substances, but do not contain structural features suggestive of toxicity; Class III stands for substances with chemical structures that permit no strong initial presumption of safety or may even suggest significant toxicity or have reactive functional groups. The implementation of the Cramer classification can be achieved using the software packages Toxtree or OECD QSAR Toolbox^2^^)^. Both are publicly available and their use is free.

The threshold values were calculated from the distribution of no-observed-adverse-effect levels (NOAELs) for each of the three Cramer structural classes, using a database of 613 chemicals representing industrial, food, environmental, agricultural, pharmaceuticals and consumer product chemicals^3^^)^. The fifth percentile NOAEL was calculated for each class and a 100-fold uncertainty factor was used to give the TTC values of 30, 9.0 and 1.5 µg/kg bw per day (or 1800, 540 and 90 µg/person per day) for Cramer classes I, II and III, respectively. The classification was further refined by the way of creating two additional classes, one for neurotoxicants including organophosphate and carbamate pesticides with a TTC value of 0.3 μg/kg bw per day (18 µg/person per day) and another class for potential DNA-reactive mutagens and/or carcinogens with a TTC value of 0.0025 μg/kg bw per day (0.15 μg/person per day). Since the original publication by Munro^3^^,^^4^^)^, several initiatives have confirmed the validity of the original TTC values using additional data sources and have confirmed, from a human health perspective, that the TTC approach is a conservative approach^5^^–^^8^^)^. These include the COSMOS database, an inventory of cosmetic substances co-funded by the European commission and cosmetics Europe^9^^)^, the RepDose database, commercial chemicals developed by the Fraunhofer Institute of Toxicology & Experimental Medicine (ITEM), Germany^10^^)^, EFSA’s OpenFoodTox dataset^11^^)^, and Hazard Evaluation Support System (HESS) database^12^^)^.

Threshold of regulation policy was first introduced in 1995 for food-contact materials in US FDA^13^^)^. TTC approach depends on the evaluation of structures in database that are used to derive the respective TTC value of the target molecule. Thus, substances such as inorganic substances, proteins, nanomaterials, etc., which are not represented in the database are outside of the domain of applicability of the TTC approach. Furthermore, some substances with special properties, such as high potency carcinogens, steroids, substances with potential bioaccumulations, etc., are also excluded from the TTC approach.

The TTC approach is currently used by several international and European bodies, e.g. European Food Safety Authority (EFSA), European Chemicals Agency (ECHA), European Medicines Agency (EMA), Joint FAO/WHO Expert Committee on Food Additives (JECFA), the non-food Scientific Committees of the European Commission (dealing issues on non-food materials like cosmetics and textile). The JECFA has been applying the TTC approach in their safety assessment of flavorings since the 1990’s. Within EFSA, the TTC approach has been used when the substances under consideration do not fall under any EU legislation that requires submission of toxicity data. Examples of the use of the TTC approach by EFSA include the evaluation of flavoring substances in food, impurities, metabolites and degradation products of food additives, pharmacologically active substances present in food of animal origin, some metabolites and degradation products of plant protection products in the context of residue definition for risk assessment, the derivation of ‘maximum acceptable feed concentrations’ for flavoring additives based on default values for feed consumption and the development of the criteria for the safety evaluation of mechanical processes to produce recycled polyethylene terephthalate (PET) intended to be used for manufacture of materials and articles in contact with food. To facilitate and harmonize the use of the TTC approach in its different sectors, EFSA’s Scientific Committee has recently developed a cross-cutting guidance document on the use of the TTC approach for food safety assessment that will include a decision tree supported by a step-by-step approach.

### 3-2 Application of the Concept of TTC Approach in the Safety Evaluation of Food Contact Materials in Japan

In Japan, food utensils, containers and packaging (UCP) have long been regulated under the Food Sanitation Act. Under this regulation, the use of substances is restricted in a way of individual specifications and standards stipulated by government (negative list system). In this system, government has no means to regulate substances whose use is not permitted outside Japan unless otherwise set the individual specifications and standards.

In addition to regulations based on this negative list, industry groups dealing with related chemicals have been implemented their own voluntary regulatory frameworks for food contact materials (FCM). There are lists of substances, which are judged to be feasible to use in accordance with their voluntary standards (voluntary list system). Although the voluntary management of FCM continued to function to certain extents, business operators, who do not affiliate to any of chemical industry group, fall outside such regulatory frameworks.

In 2018, the Ministry of Health, Labour and Welfare (MHLW) decided to adopt a new national Positive List system for FCM of synthetic resins in a few years, in order to impose restriction on substances that may be used in raw materials of food utensils, containers and packaging materials. In response to this decision, FSCJ is developing a new risk assessment scheme. In the EU and the US systems, distinct levels of toxicity data are required for inclusion in the Positive List according to the degree of migration of the food contact material into foods. Since FCMs are not intentionally added to foods, only limited amounts are reasonably assumed to migrate from FCM to foods. Thus, FSCJ is considering to require the graded levels of toxicity data depending on the migration levels of FCM and to adopt the concept of TTC approach for developing the risk assessment guidelines. In the FSCJ draft guidelines, FCMs are classified into four classes according to their potential occurrence in food as calculated from the migration tests, and thus toxicity tests requirements would be different among the classes (less migration, less requirements).

### 3-3 Category-based Read-across Approaches, Considering Human Relevance

The demand for toxicological evaluation is increasing worldwide for large numbers of marketed but untested chemicals. On the other hand, reduced animal testing is desired for animal welfare and economic reasons. Read-across is regarded as a device to predict endpoint information for a substance of unknown toxicological property (target substance), using data of the identical endpoint from other substances (source substances) which are chosen based on the similarity in the context of structure, properties and mechanism of action. It is recognized as one of the alternative approaches to complement data gap. Many efforts in developing the read-across approach are ongoing internationally to gain more experience in its use and to promote its regulatory application.

The OECD IATA (Integrated Approaches to Testing and Assessment) Case Studies Project was initiated to increase the experience with the use of a read-across IATA through developing case studies of predictions aiming for regulatory use. Between 2015 and 2018, fifteen case studies were submitted from OECD member countries/bodies and then reviewed by the project team. Two case studies on grouping and read-across for repeated dose toxicity endpoint to apply to the hazard classification under Japanese Chemical Substances of Control Law were developed by the National Institute of Health Sciences (NIHS)^14^^,^^15^^)^, and one case study was done by the collaboration between NIHS and National Institute of Technology and Evaluation, Japan^16^^)^. Due to the difficulty in building a mechanistically transparent Quantitative Structure-Activity Relationship (QSAR) models for the complex toxicological endpoint, read-across through forming a robust group of chemicals (often referred to as a category) therefore was tested as a method to complement data gaps. The review results for one Japanese case study showed that the read-across hypothesis was well justified with strong support evidence. Of course it’s improvement is necessary to clarify the structural boundaries of categories for limiting the uncertainty of toxicity prediction. Moreover, information on human relevance was identified as a key component. Through the review of several grouping case studies, the following two areas were identified as high priority for guidance development: definition of category boundaries, and uncertainty analysis and reporting. For the former, most case studies lacked a discussion on the structural differences between the individual substances in spite of fairly well documentation of their structural similarities. For the latter, each case study contained distinct levels of uncertainties derived from limited data availability or quality. Therefore, an analysis of the underlying uncertainty would provide crews for reviewer to verify the degree of uncertainty, which might be acceptable for the specified purposes.

Read-across is conceptually simple but practically needs wide range of expertise. It takes time to gather information for preparing the cases. Transparency and reproducibility are critical. To increase confidence and decrease uncertainty of read-across prediction, the choice of category needs to be justified with reliable toxicity data, possible mechanistic information and supporting data such as toxicokinetics, *in vitro* testing, omics or related information for bridging target and source chemicals. Moreover, acceptance of uncertainty may depend on the purpose of the assessment. Developing more case studies and sharing the experiences are a promising way for expanding the use of read-across.

## 4. Toward More Accurate Extrapolation of Animal Experiment Data to Human Health Effects

### 4-1 Possible Future Linkages with Toxicological Databases of Pharmaceutical Drugs

Drug-induced liver injury (DILI) is a serious safety concern with >1,000 drugs being reported to possess the potential to cause liver injury, although for most drugs this occurs rarely. DILI is a frequent cause for regulatory action, including denied approval, “black box” warnings, and withdrawal from the market. Despite extensive safety testing during the development process, the occurrence of DILI in patients is unpredictable and its causes often remain an enigma. To address this problem, the US FDA/NCTR (National Center for Toxicological Research) has been developing the Liver Toxicity Knowledge Base (LTKB) for an enhanced assessment of DILI in drug development with emerging methodologies^17^^)^. The LTKB consists of three components: curation/generation of drug-elicited data at varying degrees of complexity; development of predictive DILI models, and a software environment making both data and models publicly available. The goal of LTKB is to develop a content-rich resource to improve an understanding of liver toxicity and ultimately for the FDA to utilize and reference when liver toxicity issues arise during various stages of the regulatory review process^17^^,^^18^^)^.

The LTKB integrates a drug’s innate properties (e.g., its chemical structure and physico-chemical properties), the toxicogenomic responses to the drug treatment, mechanistically relevant cellular endpoints from *in vitro* assays, histopathology findings, and patients’ response to drug treatment to develop a correlation of data with DILI potential. This effort additionally allows interrogation of *in vitro* versus *in vivo* data to address the predictivity of supporting *in vitro* alternatives to animal testing, an endeavor embraced by the European Union, United States, and many other countries^19^^,^^20^^)^.

Within LTKB various bioinformatics strategies have been implemented for the development of DILI models using either individual homogeneous data that reflect a single layer of biological response as well as integrated approaches based on an analysis of diverse data to produce predictive models. For example, when 164 FDA-approved oral medications were analyzed, an association of high daily doses (≥ 100 mg/day) and lipophilicity (partition coefficient, logP ≥3) with significant risk for DILI was identified; thus defining a “rule-of-two”^17^^)^. This principle was further applied and verified using an independent set of 179 oral medications, drug pairs with similar chemical structures and molecular targets but different DILI potential, and in clinical case studies with complex co-medication regimes. The “rule-of-two” is an appropriate means of estimating the risk of patients developing DILI and could help support regulatory applications. In addition, a QSAR model was developed based on approximately 500 drugs with chemical descriptors generated using the in-house software Mold2 and Decision Forest^21^^)^. Another model identified 13 side effects that collectively provided an indication for DILI and that are further translated via an *in silico* approach to develop a DILI prediction system (DILIps).

The principle may apply to other similar challenges in risk assessment of compounds beyond drugs, including food-related chemicals. The advantage of toxicological databases using pharmaceutical drugs is the availability of human health effect data from e.g. clinical trials and post marketing surveillance. Possible future application of such databases to food-related chemicals may contribute to improved human health risk assessments.

### 4-2 Integration of Internal Dosimetrics into Risk Assessment of Dietary Contaminants through Use of PBPK Modeling

For improved human risk assessment, the refinement of exposure assessment in human is also important. The formation of carcinogenic contaminants during the cooking process is a major concern for food safety worldwide. Acrylamide (AA) is produced at up to part per million levels by typical during the cooking of many common foods (e.g., potato products and coffee). Many international food safety assessments have deemed AA to be a likely human carcinogen, based on its metabolism to a genotoxic metabolite (glycidamide, GA) and its carcinogenicity in both sexes and multiple organs of two rodent species^22^^,^^23^^)^.

Benchmark dose modeling provides a statistically based means to analyze tumorigenicity data from the rodent bioassays and produce parameters that can be used for human risk assessment and public educational outreach (i.e., reference value, margin of exposure). Dietary intake assessment of AA from foods has also been conducted extensively, using measurements of AA levels in a sufficient number of important foods in conjunction with food-frequency information. Worldwide, the range of estimates for median dietary intake of AA is 0.2-1 µg/kg bw/d. A key conclusion from dietary intake modeling is that because AA is found in so many common foods, even large changes in concentration for single foods or groups of foods would likely produce only minor changes in overall population-based dietary intake. Pharmacokinetic studies conducted in rats and mice produced information about concentrations of AA and GA in blood and various tissues along with biomarkers of exposure (hemoglobin adducts with AA and GA) and effect (DNA adducts with GA). PBPK models were constructed using all available measurements from animal models and humans in order to predict internal (tissue) concentrations of AA and GA in humans, primarily using extensive measurements of hemoglobin adduct measurements in non-smoking individuals from all over the world^24^^)^. The PBPK modeling produced estimates of DNA adduct levels within human tissues for use in cancer risk assessment, by comparing them with the corresponding DNA adduct levels and tumorigenicity data from lifetime exposure in rodent bioassays. The resulting risk estimations from dietary AA exposure have been consistently interpreted by international regulatory bodies as representing a concern for human health. This body of research highlights the efficient use of different modeling techniques to produce integrated cancer risk assessments for dietary exposure to cooking carcinogens, of which AA is but one out of many.

## 5. Discussion

The methodologies discussed above are at different stages of development, ranging from a research level to an implementation level in risk assessment practice in Japan.

For example, TTC approach has been already used in the assessment of flavorings globally, such as in JECFA, EFSA and Japan, and also the concept of TTC approach is planned to be included in the forthcoming FSCJ risk assessment guideline on the FCM. Read-across has already been introduced in risk assessment for metabolites of pesticides in the EFSA guidance^25^^)^. In Japan, it is currently at a preparatory stage. FSCJ published a report on the strategies to introduce QSAR and read-across as a supporting tool in addition to conventional assessment methods^26^^)^. One of the short-term action plans is to accumulate case studies using existing read-across tools such as OECD QSAR toolbox and validate their applicability to the chemical spaces of food-related chemicals. Recent developments in Europe that have emerged from extensive testing and contributions from biological information through *in vitro* testing are most encouraging^27^^,^^28^^)^.

Improving human health risk assessment of food-related chemicals is a mutual challenge for risk assessment agencies worldwide. Cross-sectorial collaboration such as making good use of already existing extensive human data of pharmaceutical drugs will facilitate further development of useful tools for food safety risk assessors, although currently food safety has not yet benefited from our experience in the human pharmaceutical sector. Integration of internal-dosimetry based on PBPK modeling is also expected to provide more refined exposure assessment in human.

Facing with common challenges, international collaboration in the development and improvement of risk assessment methodologies would have many added values for risk assessment agencies.

Efficiency would increase because we could avoid duplicated works and build on what has already been achieved. For example, data sharing in building toxicological databases among regulatory agencies in food safety domains and even with pharmaceutical drugs domains would enlarge our common knowledge bases that we can build our work on. Collaboration would be able to enhance innovative ideas on how to solve common challenges together. For example, collaboration might facilitate discussion on how we can refine the definition of similarity of chemical substances in read-across, for improving the accuracy of toxicological estimation.

Technical capacity would be also strengthened through international collaboration. The number of scientific experts in new risk-assessment methodologies is often limited within a country. Collective efforts are thus effective to provide capacity building among international partners.

Finally, international collaboration is also effective for stakeholder’s acceptance of new methodologies. Many stakeholders are hoping that any new methodology is widely accepted as a global standard and also as scientifically valid and reliable.

Once a new methodology is recognized by risk assessment agencies as implementable, it needs to be acknowledged and accepted by wider range of different stakeholders. Such stakeholders include (1) scientific experts who conduct risk assessment for the risk assessment agencies (e.g. Scientific Panel members), (2) food industries (large enterprises, small and medium-sized enterprises) and (3) consumers/general public.

The scientific experts who are engaged in risk assessment agencies would need to have sufficient confidence in these methodologies and find some benefits over the conventional methodologies. For example, a possible idea may be to offer an opportunity to experience these methodologies in some case studies they can participate in, to further strengthen their capacity to use the new methodologies.

Regarding food industries, it would be important to identify suitable approaches for companies with different levels of knowledge and capacity in the new methodologies. For some companies who have already made financial and/or time investments in introducing new methodologies, they might already have sufficient capacity to implement these methodologies. They could find many benefits such as its efficiency. In contrast, for other companies who are not as experienced, they might perceive such a shift to new methodologies as a business risk that increases their costs or workloads. Communication between regulatory agencies and industries may be encouraged to fill in the information gap regarding different needs in accepting and introducing new methodologies.

As for general public or consumers, they are necessary to get interested in knowing about the technical details of the new methodologies. They would rather show interest to realize the implication of introducing these methodologies to the results of risk assessment. The credibility in the risk assessment agencies among consumers is one of the key factors for consumers to accept new methodologies. Transparency, in which the risk assessment agencies explain about the methodologies and scientific evidence behind their risk assessment judgment, should strengthen their credibility in risk assessment works and thereby promote the acceptance of risk assessment including the new methodologies used. At the same time, efforts should be made by regulatory agencies to further communicate with general consumers about the concept of risk-based assessment as well as the concept of uncertainty.
